# Integrated Analysis of miRNA and mRNA Expression Profiles Associated with Development of Skeletal Muscle of Jiangquan Black Pigs

**DOI:** 10.3390/genes16060701

**Published:** 2025-06-12

**Authors:** Yarui Gao, Shiyin Li, Wei Chen, Jianmin Zhang, Zhanchi Ren, Zhao Ma, Yunzhou Wang, Yongqing Zeng

**Affiliations:** 1 Shandong Provincial Key Laboratory for Livestock Germplasm Innovation & Utilization, College of Animal Science and Technology, Shandong Agricultural University, Tai’an 271018, China; 13721751403@163.com (Y.G.); garyli1121@163.com (S.L.); wchen@sdau.edu.cn (W.C.); zhangjianmin23@163.com (J.Z.); 17861218765@163.com (Z.R.); 15206902825@163.com (Z.M.); 2 Department of Veterinary Medicine, Shandong Vocational Animal Science and Veterinary College, Weifang 261061, China

**Keywords:** Jiangquan black pigs, longissimus dorsi muscle, RNA-seq, skeletal muscle, small RNA sequencing

## Abstract

Background: Hypertrophy, myogenic differentiation, and mass gain of porcine skeletal muscle are key factors in meat production efficiency, regulated by miRNAs through post-transcriptional mechanisms. This study aims to identify miRNA-mRNA pairs linked to growth and muscle development in Jiangquan Black pigs with differing average daily gains (ADGs), providing a foundation for molecular breeding in this breed. Methods: This study divided eight pigs into two groups and analyzed the skeletal muscle characteristics of Jiangquan Black pigs with different average daily weight gains using HE staining. RNA-Seq was conducted to identify differentially expressed miRNAs and mRNAs, Gene Ontology (GO) enrichment analysis and Kyoto Encyclopedia of Genes and Genomes (KEGG) pathway analysis were performed, and an integrated miRNA-mRNA regulatory network was subsequently constructed. Results: RNA sequencing analysis identified 255 differentially expressed genes (DEmRNAs, |FC| > 1.5) and 27 differentially expressed miRNAs (DE miRNAs, |FC| > 2). Bioinformatics analysis revealed 330 significantly negatively correlated miRNA-mRNA regulatory pairs, with key pathways, including the MAPK, mTOR, insulin, FoxO, Wnt, and TGF-β signaling pathways, being implicated in muscular development. Quantitative real-time PCR (qRT-PCR) validation confirmed the reliability of the sequencing data. Conclusions: Different ADGs among half-sibling Jiangquan Black pigs with the same diet may be due to the DE miRNAs and DEmRNAs related to skeletal muscle growth and development. These findings reveal the potential regulatory mechanisms of DE miRNAs and DEmRNAs in porcine skeletal muscle growth, providing valuable insights for the next steps in molecular breeding strategies for Jiangquan Black pigs.

## 1. Introduction

Pigs are one of the most important sources of animal protein for human consumption, as their skeletal muscle has a large protein composition [1]. The growth and development of skeletal muscle directly affect the quality and yield of pork [2]. Studying the molecular mechanisms of growth and development of porcine skeletal muscle is crucial to improving growth rates in pigs [3]. The development of skeletal muscle is not only influenced by factors such as genes, hormones, and nutrition but also regulated by the complex action of miRNAs. miRNAs are a group of endogenous, non-coding small single-stranded RNAs that are widely present in living organisms and highly conserved during evolution [4]. They exert their regulatory effects by binding to the 3′ UTR region of target mRNAs, inhibiting gene expression, leading to suppression of protein translation or mRNA degradation [5]. As key post-transcriptional regulators of gene expression, miRNAs play a crucial role in the complex, dynamic network involved in organismal development.

In recent years, numerous studies have been published on the regulation of skeletal muscle growth and development by miRNAs. Several muscle-specific miRNAs, such as miR-1, miR-133, and miR-206, have been identified, which are expressed specifically and at high levels in muscle tissues [6]. They are key regulatory factors in skeletal muscle development, and their expression is controlled by transcription factors such as *HDAC4*, *SRF*, and *Pax7*. miR-1 enhances muscle development by suppressing the post-transcriptional expression of goat *HDAC4* [7]. miR-133 promotes myoblast proliferation by inhibiting serum response factor (*SRF*) [8], while miR-206 directly inhibits *Pax7* expression, leading to the exit of myoblasts from the proliferation and cell cycle stages, followed by their entry into the differentiation phase [9]. Additionally, miR-195 and miR-497 target and suppress the expression of *cyclin D2* and cyclin-dependent kinase 25a/b, further inducing cell cycle exit to maintain the quiescent state [10]. miR-34c reduces the proliferation of satellite cells in pig skeletal muscle and enhances their differentiation through the suppression of *Notch1* expression [11]. miR-489 maintains the quiescent state of muscle satellite cells by targeting and inhibiting the oncogene *Dek* [12]. These results highlight the essential role of miRNAs in skeletal muscle development.

Currently, studies on miRNAs in the muscles of various animal species primarily concentrate on comparing animals with diverse genetic backgrounds, while comparative research involving half-siblings remains relatively scarce. In our previous research, we identified and functionally predicted circular RNAs, long non-coding RNAs, and mRNAs linked to growth traits and skeletal muscle development in Duroc pigs, considering variations in average daily gain and half-sibling relationships [13,14]. The half-sibling design enhances genetic diversity, helping to better distinguish the impact of genetic and environmental factors. The Jiangquan Black pig is a newly developed fast-growing, high-quality meat pig breed, created by using the local Shandong Yimeng Black pig as breeding material and introducing lean-type pig breeds such as Duroc through crossbreeding and generations of selective breeding. In this study, half-sibling Jiangquan Black pigs were divided into the F group (H-ADG) and the S group (L-ADG) to identify candidate miRNA-mRNA gene pairs related to skeletal muscle development, providing a theoretical basis for the molecular breeding of Jiangquan Black pigs in the next phase.

## 2. Materials and Methods

### 2.1. Ethics Statement

All procedures for the care and treatment of animals were conducted in complete accordance with the guidelines and regulations set forth by the Animal Ethics Committee at Shandong Agricultural University in China (Approval SDAUA-2022-112).

### 2.2. Animals

Based on the current measurement data, 50 Jiangquan sows with fast growth rates and high average daily gains were selected from the herd. The environmental conditions for all pigs fully complied with animal welfare standards. We selected eight pigs with half-sibling relationships from a group of 50 Jiangquan Black pigs, and divided these eight pigs into two groups, the F group (a high average daily gain group, 597.18 g) and the S group (a low average daily gain group, 525.47 g), based on their average daily weight gains. Subsequently, samples of the longissimus dorsi muscle (LDM) tissue were collected from these pigs and immediately frozen in liquid nitrogen, and total RNA was extracted.

### 2.3. Paraffin Section and H&E Staining

Hematoxylin and eosin (H&E) staining was performed using a standard protocol. In brief, tissue samples were fixed in 4% paraformaldehyde, followed by routine dehydration and embedding. After the standard preparation of tissue sections, the paraffin was removed. The sections were stained with hematoxylin and eosin for a duration of 8–10 min, followed by a 10 min wash with tap water. Images were obtained with an inverted fluorescence microscope (OLYMPUS, Tokyo, Japan), and the skeletal muscle fiber diameter and cross-sectional area were quantified using Image J software (National Institutes of Health, Bethesda, MD, USA).

### 2.4. RNA Extraction, Library Construction, and RNA Sequencing

Library construction and deep sequencing were performed by Genedno Biotechnology Co., Ltd. (Guangzhou, China). Total RNA, including both small RNA and mRNA, was extracted from LDM tissue samples. For comprehensive transcriptomic analysis, we conducted parallel small RNA and mRNA extractions from longissimus dorsi muscle tissue samples derived from both the F and S groups. Eight small RNA libraries and eight mRNA libraries were constructed, with four biological replicates performed for each group. RNA integrity was assessed using the Agilent 2100 Bioanalyzer (Agilent Technologies, Santa Clara, CA, USA).

For mRNA library preparation, rRNA was removed using the Ribo-Zero™ (San Diego, CA, USA) Gold rRNA removal kit, followed by mRNA fragmentation. First-strand cDNA synthesis was performed using random hexamer primers, and second-strand cDNA synthesis was carried out with DNA polymerase I, RNase H, and a dNTP mixture containing dUTP. The resulting double-stranded cDNA was purified and subjected to end repair, 3′ adenylation, and ligation with Illumina adaptors. Strand specificity was achieved through uracil-DNA glycosylase (UDG) treatment during PCR amplification. The libraries were size-selected (300–500 bp) and quantified by qPCR, followed by sequencing on the Illumina HiSeq 4000 platform (San Diego, CA, USA).

For small RNA library construction, total RNA was extracted using TRIzol reagent (Invitrogen, Carlsbad, CA, USA) and size-separated on 15% denaturing polyacrylamide gel electrophoresis (PAGE) to select fragments in the 18–30 nt range. Small RNA was ligated with 3′ and 5′ adaptors, followed by intermediate size selection (36–44 nt). The ligation products were reverse-transcribed and amplified by PCR, and the resulting 140–160 bp cDNA fragments were purified to construct the library. After quantification and quality control, the libraries were sequenced using the Illumina NovaSeq 6000 platform with paired-end reads.

### 2.5. Mapping and Assembly of Sequenced RNA Data

Raw sequencing data underwent quality control using fastp (v0.12.4) [14] to remove reads with N and base mass values (Q) below 20. Data quality was assessed using FastQC (v0.11.9). Clean reads were aligned to the Sus scrofa v11.1 reference genome (Ensembl) following Hisat2 (v2.2.1) index construction. SAM files were processed to BAM format using Samtools (v1.15). Transcript quantification (HTseq v2.0.1) and assembly (StringTie v2.2.1) generated eight GTF files, subsequently merged into a non-redundant composite. miRNA analysis utilized miRBase v22.1 references with mirdeep2 (v0.1.3) for alignment and quantification.

### 2.6. Analysis of Differentially Expressed mRNAs and miRNAs

RNA sequencing data were processed using the FPKM (Fragments Per Kilobase of transcript per Million mapped reads) normalization method to account for transcript length and sequencing depth variations. For miRNA analysis, expression levels were normalized by transcripts per million (TPM), and differential expression was assessed using the DEGSeq R package. We used *p*-value < 0.05 and |fold change| > 2 as criteria to identify DE miRNAs. The screening criteria for DEmRNAs were defined as |fold change| > 1.5 with a statistical significance threshold of *p*-value < 0.05.

### 2.7. Functional Enrichment Analysis

Gene Ontology (GO) [15] functional annotation and enrichment analysis of DEmRNAs and target genes of DE miRNAs were performed using the DAVID bioinformatics platform (v6.8; https://david.ncifcrf.gov/, accessed on 10 April 2024), with comprehensive evaluation of biological processes (BPs), cellular components (CCs), and molecular functions (MFs). KEGG [16] pathway enrichment analysis was conducted via the KOBAS 3.0 system (KEGG Orthology Based Annotation System; http://bioinfo.org/kobas accessed on 10 April 2024), with the statistical significance threshold set at *p*-value < 0.05. Biological pathway visualization and interpretation were subsequently performed using the Reactome pathway analysis toolkit.

### 2.8. Protein–Protein Interaction (PPI) Network Establishment

The protein interactions encoded by the DEmRNAs were assessed using the STRING v11.0 online tool to build the PPI network, which was then visualized with Cytoscape software (Version 3.2.0). DEmRNA-encoded proteins were designated as nodal elements within the network, while their functional interactions were represented as edges. The resulting PPI network was computationally visualized using Cytoscape software.

### 2.9. Target Gene Prediction of Differentially Expressed miRNAs

To predict the target genes of DE miRNAs, we utilized three target gene prediction platforms: Miranda, RNAhybrid, and TargetScan. These tools were employed to identify the target genes of DE miRNAs and to explore their intersections with DEmRNAs [17]. For the predictions, 2–8 nucleotide sequences, derived from the 5′ end of small RNAs, were selected as seed sequences to interact with the 3′-UTRs of transcripts. Considering the repressive role of miRNAs, we concentrated on calculating the proportion of negatively correlated miRNA-mRNA pairs. Specifically, we focused on those pairs where up-regulated miRNAs were associated with down-regulated mRNAs, or up-regulated mRNAs were linked to down-regulated miRNAs, in the miRNA-mRNA target pairs predicted by Miranda, RNAhybrid, and TargetScan. Finally, we established a potential regulatory network linking DE miRNAs and DEmRNAs through Cytoscape analysis (Version 3.2.0).

### 2.10. Quantitative Real-Time Polymerase Chain Reaction Analysis

Total RNA was isolated from LDMs with the use of Trizol reagent. cDNA for mRNA was synthesized using random primers, while cDNA for miRNA was synthesized by tailing A. Quantitative real-time PCR was conducted using SupRealQ Purple Universal SYBR qPCR Master Mix (Q412, Vazyme, Nanjing, China), and amplification was detected using the Roche LightCycler 96 system (Roche, Basel, Switzerland). The expression levels of mRNA were normalized to GAPDH as the internal control, while U6 was used for normalizing miRNA expression, with all reactions conducted in triplicate. The primers utilized for qPCR are provided in Supplementary Table S1. The 2^−∆∆CT^ method was applied to analyze the data obtained from the experiments.

### 2.11. Statistical Analysis

Statistical analysis was conducted using SPSS 22.0 (IBM, Chicago, IL, USA). Independent sample *t*-tests were conducted to compare histological parameters of muscle fiber characteristics between groups, with results presented as mean ± SD, and *p* < 0.05 was considered statistically significant. Data visualization was generated using GraphPad Prism 8.0.1 (GraphPad Software, San Diego, CA, USA).

## 3. Results

### 3.1. Comparison of Morphological Characteristics of Muscle Fibers

**Figure 1:**
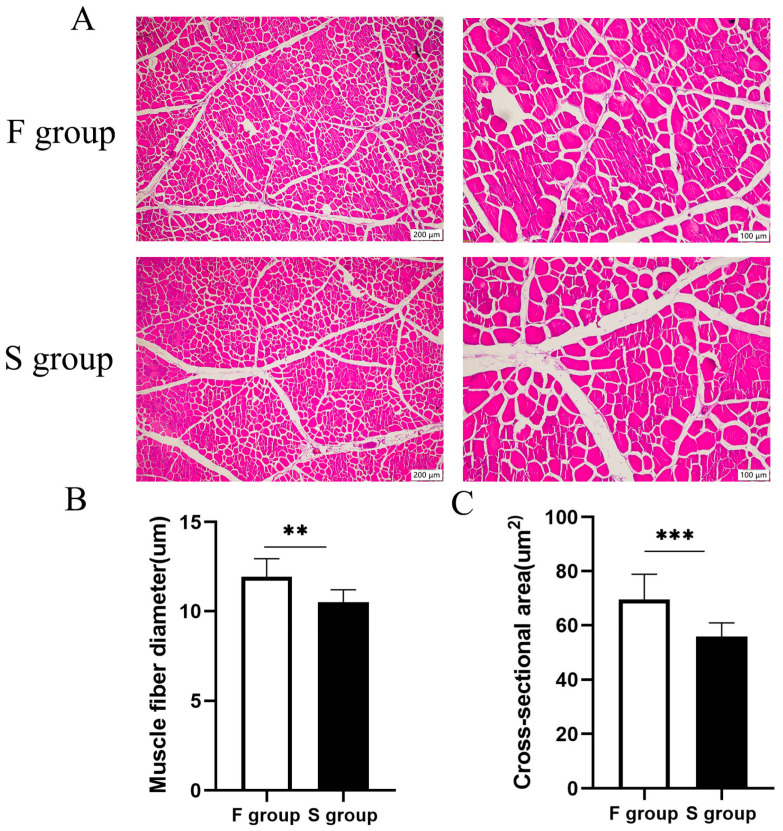
Morphology differences in LDMs of Jiangquan Black pigs. (**A**) The morphology of skeletal muscles in the F group and S group was measured by HE staining (scale bar = 200 um). (**B**) Analysis of myofiber diameter and (**C**) cross-sectional area was conducted. Vertical bars represent mean ± standard error of mean (*n* = 10). ** *p* < 0.01, *** *p* < 0.001.

To compare the morphological characteristics of muscle fibers between the F group and S group, HE staining was performed for histological analysis (Figure 1A). The results demonstrated that the F group had a significantly larger myofiber diameter (Figure 1B) and cross-sectional area (Figure 1C) compared to the S group, indicating that the F group had significantly more intense skeletal muscle development than the S group.

### 3.2. Identification of Differentially Expressed mRNAs

**Figure 2:**
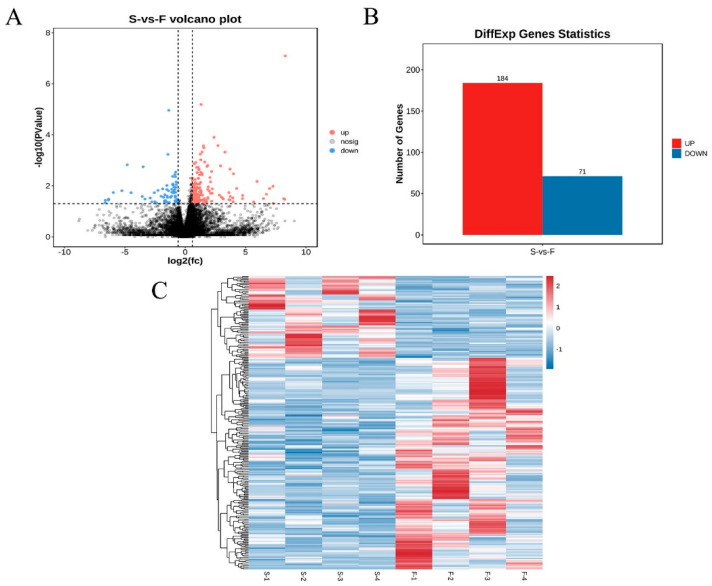
Statistics and heatmap analyses of DEmRNAs. (**A**) Volcano plot of DEmRNAs. (**B**) Column chart of DEmRNAs. (**C**) Heatmap of DEmRNAs.

Following the removal of low-quality reads and adapter sequences, high-quality clean data were retained at a rate of 99.62% (F group) and 99.58% (S group). The GC content of the clean reads ranged from 55.90% to 57.96%, with Q20 and Q30 quality scores higher than 92.21% and 93.14% (Table 1), respectively. The results showed that the sequencing data had high quality and reliability. A total of 255 DEmRNAs were identified, showing that 184 genes were up-regulated and 71 genes were down-regulated (Figure 2A,B, Supplementary Table S2). The differential expression patterns of mRNAs were then analyzed through heatmap generation (Figure 2C). *PAK1*, *MYL10*, *IGFBP5*, and *BMP5* were highly expressed and *MYOM3* was lowly expressed in the F group.

### 3.3. Functional Enrichment of Differentially Expressed mRNAs

**Figure 3:**
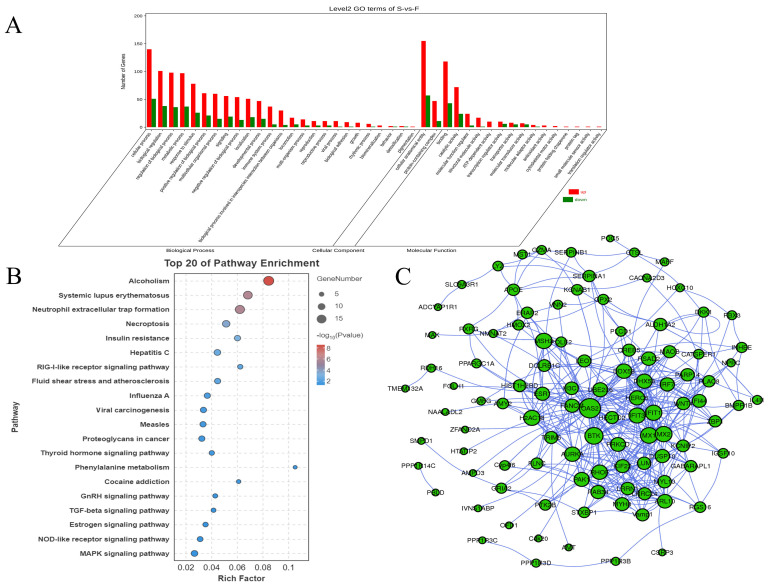
Functional enrichment analysis of DEmRNAs and protein–protein interaction (PPI) network. (**A**) GO term enrichment analysis of DEmRNAs. (**B**) KEGG analysis of DEmRNAs. (**C**) Protein–protein interaction (PPI) network of differentially expressed genes.

To better understand the functional roles of the DEmRNAs, we conducted GO and KEGG enrichment analysis, identifying 42 significantly enriched GO terms (Supplementary Table S4) and 246 significantly enriched KEGG pathways (Supplementary Table S5). GO annotation categorizes genes into three main divisions: cellular component (CC), molecular function (MF), and biological process (BP). In this study, the target genes of DE miRNAs were significantly enriched in 25 BP, 15 MF, and 2 CC categories. The results demonstrated that the DEmRNAs were significantly enriched in GO functional categories, including metabolic processes, cellular processes, regulation of biological processes, growth, multicellular organismal processes, catalytic activity, and cell motility (Figure 3A). Among the top 20 KEGG pathways, GnRH and TGF-β signaling pathways were identified as skeletal muscle development-related pathways (Figure 3B). Furthermore, within all 246 KEGG pathways analyzed, 7 pathways demonstrated significant associations with skeletal muscle development: GnRH, TGF-β, MAPK, insulin, AMPK, FoxO, and mTOR signaling pathways. Therefore, these genes, which are enriched in pathways related to skeletal muscle development, could be crucial in regulating the growth and development of skeletal muscle in Jiangquan Black pigs.

### 3.4. DEmRNA-Mediated Protein–Protein Interaction Network Analysis

Protein interaction network maps were created to better understand the relationships between differentially genes. By using the STRING online database and Cytoscape visualization tool, 255 DEmRNAs encoding proteins were selected for constructing the PPI network. PPI network analysis revealed that 104 out of 255 DEmRNA-encoded proteins demonstrated significant protein–protein interactions. The analysis shows that OAS2, H2AC18, MX1, PAK1, MYH8, and MYL10 are key hubs (Figure 3C).

### 3.5. Identification of Differentially Expressed miRNAs

**Figure 4:**
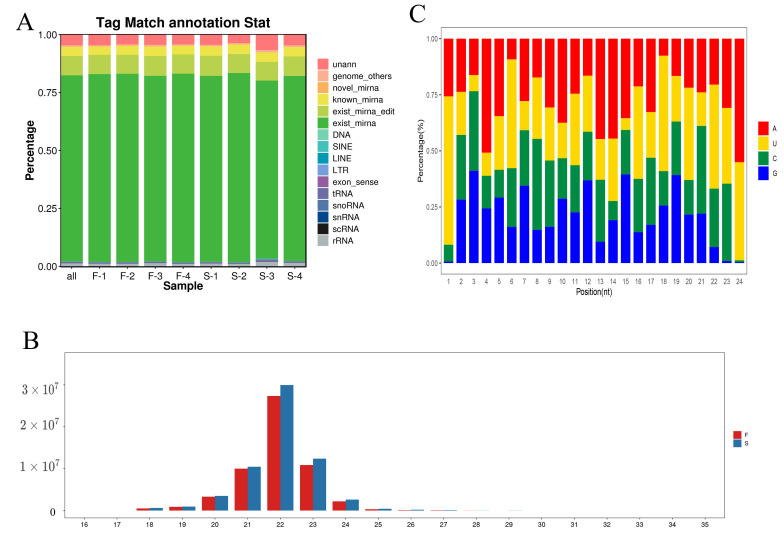
Characterization of small RNAs. (**A**) Characteristics of known and novel miRNAs. (**B**) Percentage distribution of all small mRNA sequence lengths. (**C**) First nucleotide bias of miRNAs.

**Figure 5:**
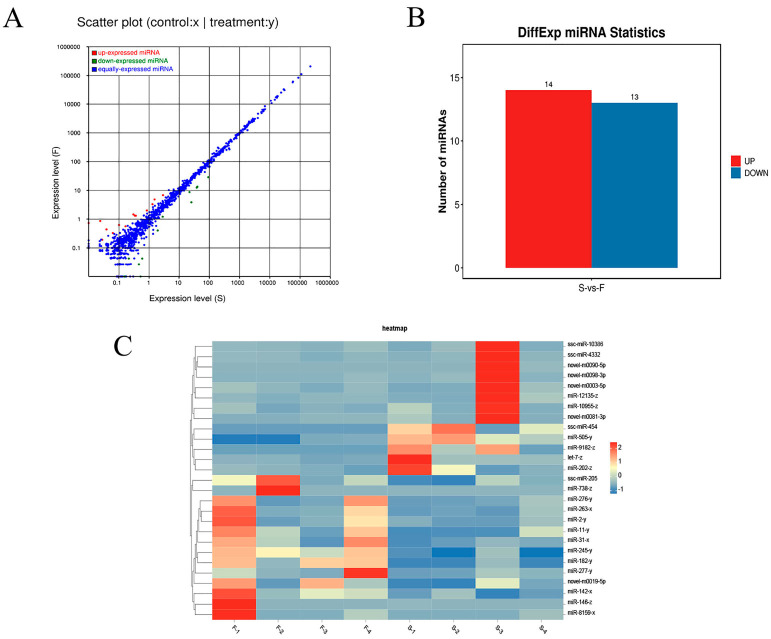
Analysis of DE miRNAs. (**A**) Scatter plot of DE miRNAs. (**B**) Column chart of DE miRNAs. (**C**) Heatmap of DE miRNAs.

The eight libraries in this study were processed to eliminate the reads containing adapters (about 0.56%) and low-quality reads (about 1.18%). As a result, about 98.26% of the raw data reached the quality control standards and became qualified data for subsequent small RNA analysis (Supplementary Table S3). Quantitative small RNA analysis revealed that the percentage of miRNAs was no less than 80.05% and 76.84% of the total small RNAs in the LDMs of Jiangquan Black pigs for groups F and S (Figure 4A, Supplementary Table S3), respectively. The majority of miRNAs fall in the 18–24 nt length range, with 22 nt miRNAs being the most abundant in both groups (Figure 4B), accounting for 48.88% in group F and 49.16% in group S (Supplementary Table S3). Furthermore, the analysis also showed a clear preference in the distribution of miRNA bases in the LDMs of Jiangquan Black pigs. Most miRNAs began with either A or U bases, with U being the most common, representing approximately 28.19% of all bases (Figure 4C). A total of 27 DE miRNAs were identified (Figure 5A,B). Compared to the S group, 14 miRNAs were up-regulated and 13 miRNAs were down-regulated in the LDMs of Jiangquan Black pigs in group F (Figure 5C, Supplementary Table S3).

### 3.6. Functional Enrichment of Target Genes of Differentially Expressed miRNAs

**Figure 6:**
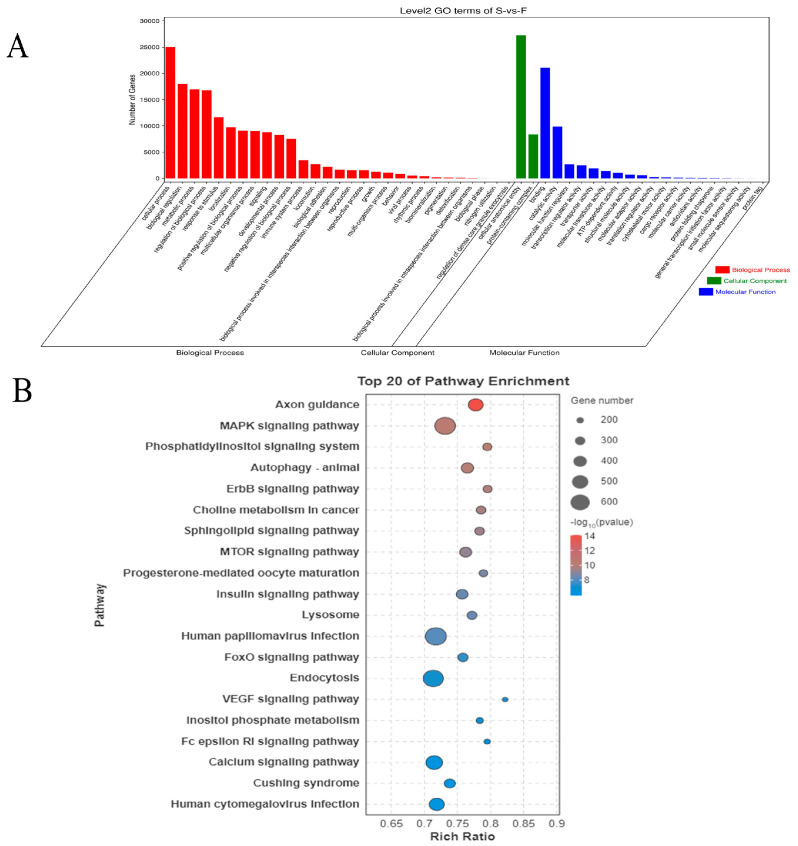
Functional enrichment analysis of target genes of DE miRNAs. (**A**) Gene Ontology term enrichment analysis of target genes of DE miRNAs. (**B**) KEGG analysis of target genes of DE miRNAs.

To explore the differential expression patterns of target genes of DE miRNAs between the F and S groups, enrichment analysis of the target genes of DE miRNAs was performed using the Gene Ontology (GO) database. The results showed significant enrichment in 29 BP categories, 19 MF categories, and 2 CC categories (Figure 6A, Supplementary Table S4). The results demonstrated that the target genes of DE miRNAs were significantly enriched in GO functional categories, including biological process involved in interspecies interaction between organisms, multi-organism process, developmental process, growth, metabolic process, and cellular process.

A total of 90,155 target genes of DE miRNAs were significantly enriched in 356 signaling pathways (Supplementary Table S5). The top five KEGG pathways included axon guidance, MAPK signaling pathway, phosphatidylinositol signaling system, autophagy-animal, and ErbB signaling pathway (Figure 6B). Among the 356 KEGG pathways related to skeletal muscle development were the MAPK, mTOR, insulin, FoxO, and Wnt signaling pathways.

### 3.7. Regulatory Network Analysis of DE miRNAs-DEmRNAs

**Figure 7:**
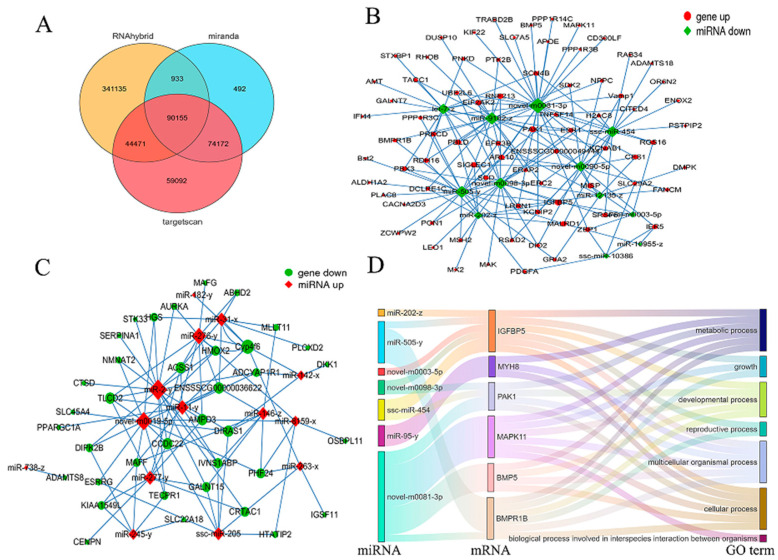
Interaction network of DE miRNAs-DEmRNAs. (**A**) Venn diagram of target genes of DE miRNAs. (**B**) Down-regulated miRNAs versus up-regulated mRNAs. (**C**) Up-regulated miRNAs versus down-regulated mRNAs. (**D**) Sankey diagram of significantly enriched GO terms for DE miRNA target genes associated with skeletal muscle growth and development in Jiangquan Black pigs.

To investigate the regulatory roles of miRNAs and mRNAs in the skeletal muscle development of Jiangquan Black pigs, we further predicted the potential target genes of the DE miRNAs using RNAhybrid, Miranda, and TargetScan. A total of 90,155 overlapping genes were identified across the three databases (Figure 7A). In addition, a coexpression regulatory network of DE miRNA-DEmRNAs was set up on the basis of the target gene prediction results (Figure 7B,C), and 24 important DE miRNAs were identified. The findings showed that DE miRNAs have target relationships with several genes linked to skeletal muscle development, such as *MYH8*, *IGFBP5*, *PAK1*, *MAPK11*, *BMP5*, and *BMPR1B*. For instance, miR-202-z targets the *IGFBP5* gene, miR-95-y targets the *MYH8* gene, ssc-miR-454 targets the *PAK1* gene, and novel-m0081-3p targets the *MAPK11* gene, suggesting that miRNA-mRNA regulation may play a role in the growth and development of skeletal muscles in Jiangquan Black pigs. Additionally, a Sankey diagram was used to visually illustrate the relationships between the DE miRNAs, their target genes, and the significantly enriched GO terms associated with skeletal muscle development (Figure 7D).

### 3.8. Validation of Differentially Expressed Genes by Quantitative Real-Time Polymerase Chain Reaction

**Figure 8:**
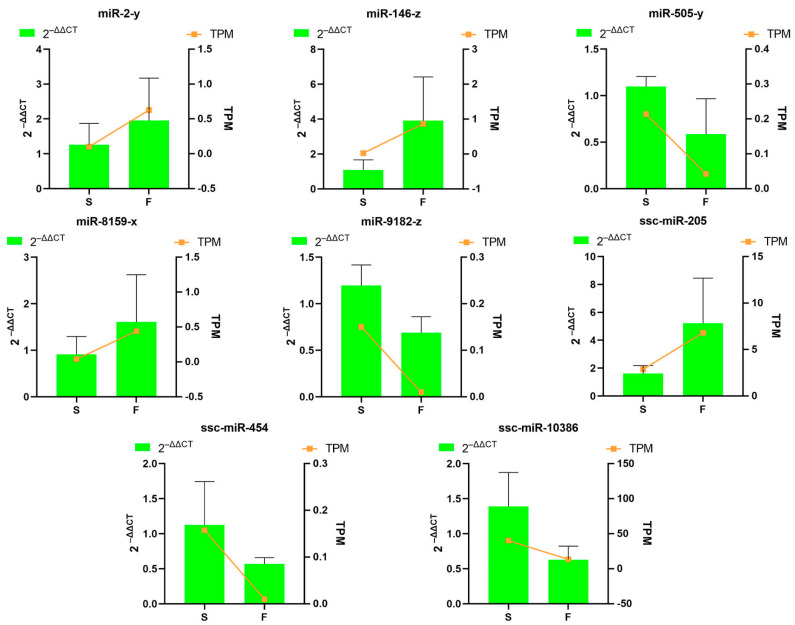
Comparison of miRNA expression levels using RNA-Seq and RT-qPCR. RT-qPCR data are reported as mean ± SD, with 2^−∆∆Ct^ representing the RT-qPCR result. The TPM value is utilized to represent the RNA-Seq result.

**Figure 9:**
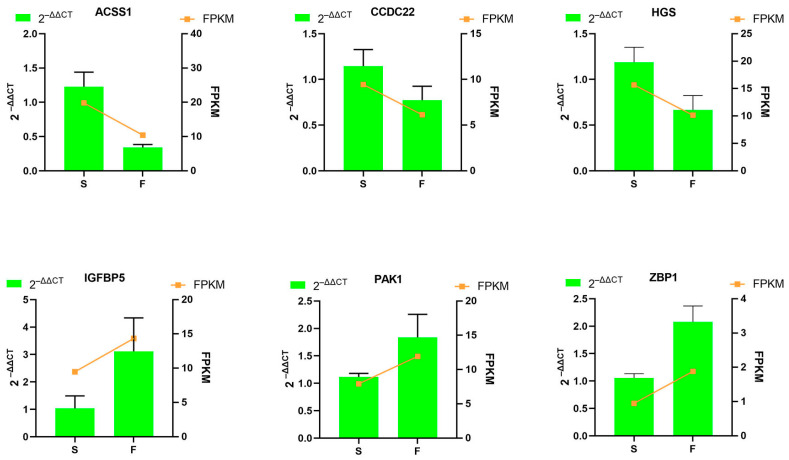
Comparison of mRNA expression levels using RNA-Seq and RT-qPCR. RT-qPCR data are reported as mean ± SD, with 2^−∆∆Ct^ representing the RT-qPCR result. The FPKM value is utilized to represent the RNA-Seq result.

To validate the DEmRNAs and DE miRNAs identified by RNA-seq analysis, we selected eight DE miRNAs (miR-146-z, ssc-miR-205, miR-2-y, miR-8159-x, ssc-miR-454, miR-9182-z, ssc-miR-10386, and miR-505-y) and six DEmRNAs (PAK1, ZBP1, IGFBP5, HGS, CCDC22, and ACSS1) for qRT-PCR confirmation. The expression profiles of DEmiRNAs validated by qRT-PCR showed high consistency with RNA-seq data (Figure 8). More importantly, the DEmRNAs validation results further supported this conclusion (Figure 9).

## 4. Discussion

Skeletal muscle constitutes a predominant portion of the body composition in livestock, representing approximately 40% of the total body weight in meat-producing species [18]. As the primary determinant of carcass yield and meat quality, skeletal muscle growth represents one of the most economically significant traits in swine production. Myofiber characteristics (including diameter and cross-sectional area) serve as critical histological indicators for assessing both muscle development dynamics and ultimate meat quality parameters. The results of HE staining in this research showed a significantly larger myofiber diameter and cross-sectional area in group F compared to group S. These pronounced morphological differences may be critically associated with the observed growth rate variations between the two groups. We performed a comprehensive analysis of miRNA-mRNA expression profiles in Jiangquan Black pigs with high and low average daily weight gains using high-throughput sequencing, identifying candidate genes linked to skeletal muscle development in the longissimus dorsi muscle.

The GO enrichment analysis of these candidate genes revealed their significant involvement in several key molecular functions, including biological processes related to interspecies interactions, developmental processes, reproductive processes, growth, and metabolic pathways. These genes may play a critical role in the growth and development of skeletal muscle. It was observed that *MYOM3* was highly expressed in the S group, whereas *MYH8*, *MYL10*, *PAK1*, *IGFBP5*, *BMP5*, and *BMPR1B* exhibited elevated expression in the F group. Inhibition of *MYOM3* was shown to suppress the proliferation of ovine myoblasts while promoting their differentiation into myocytes [19,20]. The regulation of muscle growth and development is significantly influenced by *MYL10*, while *MYH8* serves as a marker of muscle regeneration [21,22]. The expression of *MYH8* predominates in the initial phases of muscle development [23]. IGFBP5 belongs to the family of insulin-like growth factor-binding proteins (IGFBPs) [24]. During myogenic cell differentiation, the presence of IGFBPs inhibits muscle cell differentiation. By interfering with IGF expression, these binding proteins ultimately suppress protein production [25]. Furthermore, overexpression of *IGFBP5* can promote or inhibit IGF-mediated myogenic cell differentiation or survival by either promoting or blocking the action of *IGF* [26]. *PAK1* promotes the increase in skeletal muscle mass induced by myostatin inhibition [27]. Furthermore, genes such as *PAK1*, *MAPK7*, and *MAPK11* are enriched in the MAPK signaling pathway, while *BMP5* is enriched in the TGF-β signaling pathway. The sequencing of the longissimus dorsi muscle at different developmental stages of Jinfen White pigs identified the MAPK signaling pathway [28], while the FoxO signaling pathway was also detected in studies of Duroc pigs with varying growth rates [13]. Previous studies have confirmed that potential targets of DE lncRNAs during porcine embryonic development are primarily enriched in the TGF-β signaling pathway [29], which also plays a crucial role in muscle development in mice [30]. These genes may regulate the growth and development of skeletal muscle through their respective signaling pathways.

miRNAs have been highlighted as key regulatory factors in muscle development and play a crucial role in the development of skeletal muscle. Previous studies have shown that miR-143 and miR-145 can regulate genes such as *KLF4* and *TGF-β*, participating in the development and repair of muscle cells [31]. Overexpression of bta-miR-181d and bta-miR-196a in Qin Chuan cattle myocytes inhibits proliferation and apoptosis while promoting myogenesis, potentially through the modulation of key protein phosphorylation in the PI3K-Akt and MAPK-ERK signaling pathways [32]. miR-206 is a classic muscle-specific miRNA that plays an important role in the development and repair of skeletal muscle [33]. Studies have found that miR-206, specifically expressed in skeletal muscle [34], promotes the differentiation of muscle satellite cells by targeting *Pax7*, a marker gene for these cells [35]. In this study, we identified 27 DE miRNAs, among which miR-202-z, miR-182, miR-205, and miR-142-5p may play a potential role in the regulatory mechanisms underlying skeletal muscle development. miR-202-z exhibits different expression patterns in the skeletal muscle of Wuzhishan pigs and Landrace pigs, and may be involved in the proliferation and differentiation of muscle cells, thereby influencing skeletal muscle development [36]. miR-182 can promote the differentiation of primary bovine myoblasts by negatively regulating the expression of *CFL1* [37]. In addition, miR-205 inhibits myoblast fusion by targeting Myomaker expression [38]. And miR-142-5p targets *FOXO3*, enhancing growth-related gene expression and regulating skeletal muscle growth in chickens [39]. Five novel and functionally unknown DE miRNAs were identified in our study. Further research is needed to explore their functions and regulatory mechanisms. We also found that the target genes of these differentially expressed miRNAs are significantly enriched in several signaling pathways such as mTOR, MAPK, and FoxO. Research indicates that miR-100-5p modulates the *Trib2* gene during myogenic differentiation to regulate the mTOR signaling pathway [40]. And miR-3525 regulates the proliferation and differentiation of skeletal muscle satellite cells by targeting *PDLIM3* through the MAPK signaling pathway [41]. These findings suggest that DE miRNAs may play a crucial role in the myogenic differentiation and cell proliferation of skeletal muscle cells by regulating signaling pathways associated with skeletal muscle development.

We further constructed an mRNA-miRNA regulatory network and a Sankey diagram to explore the regulatory functions of candidate miRNAs and mRNAs in skeletal muscle development. Since miRNAs exert their functions by inhibiting mRNA transcription or translation, we only constructed the miRNA-mRNA network with negative regulatory relationships. *IGFBP5* is co-regulated by ssc-miR-454, miR-202-z, miR-505-y, novel-m0003-5p, and novel-m0098-3p, playing a pivotal role in skeletal muscle development in both pigs and cattle [42,43]. *ACSS1*, which is co-regulated by miR-11-y, miR-276-y, miR-277-y, miR-2-y, and miR-31-y, serves as a crucial enzyme in skeletal muscle energy metabolism and is closely associated with carcass traits and meat quality [44]. It primarily participates in the conversion of free acetate from both the mitochondria and cytoplasm into acetyl-CoA, which subsequently contributes to fatty acid synthesis [45]. These studies indicate that the miRNA-mRNA regulatory network established in this research is a key player in the regulation of skeletal muscle development in pigs. However, further research is needed to clarify the regulatory mechanisms of these miRNAs, their target genes, and the complex interaction networks involved in pig skeletal muscle development.

## 5. Conclusions

In this study, we identified 27 DE miRNAs and 255 DEmRNAs. The majority of the DEmRNAs and target genes of DE miRNAs are enriched in signaling pathways such as MAPK, mTOR, FoXO, and Wnt. Consequently, these DE miRNAs may play a pivotal role in growth traits. Through integrated miRNA-mRNA analysis, the interactions of miR-202-z-*IGFBP5*, miR-95-y-*MYH8*, and ssc-miR-454-*PAK1* may potentially contribute to the growth and development of skeletal muscle. The findings of this study provide significant reference value for muscle development research and molecular breeding. However, it is important to note that the regulatory mechanism of the miRNA-mRNA interaction in the development of porcine skeletal muscle requires further validation and analysis through cellular experiments in the next phase, as current experiments are insufficient to draw definitive conclusions. We plan to address these gaps in future studies.

## Figures and Tables

**Table 1:** Statistics for sequencing read quality control.

Sample	Clean Data (bp)	Q20 (%)	Q30 (%)	N (%)	GC (%)
F-1	14,169,221,396	13,820,127,751 (97.54%)	13,247,777,202 (93.50%)	19,211 (0.00%)	7,920,814,869 (55.90%)
F-2	15,455,529,768	15,078,387,256 (97.56%)	14,478,846,918 (93.68%)	194,491 (0.00%)	8,879,556,177 (57.45%)
F-3	14,920,893,046	14,571,191,202 (97.66%)	14,003,985,159 (93.85%)	18,745 (0.00%)	8,647,668,938 (57.96%)
F-4	15,350,988,150	15,024,061,510 (97.87%)	14,489,584,711 (94.39%)	193,383 (0.00%)	8,869,412,762 (57.78%)
S-1	15,442,610,918	15,051,361,427 (97.47%)	14,432,699,483 (93.46%)	195,029 (0.00%)	8,692,984,968 (56.29%)
S-2	14,437,471,550	14,082,357,667 (97.54%)	13,524,312,905 (93.68%)	181,158 (0.00%)	8,391,017,741 (58.12%)
S-3	14,095,330,254	13,752,865,783 (97.57%)	13,201,990,565 (93.66%)	175,519 (0.00%)	7,982,128,285 (56.63%)
S-4	14,017,378,016	13,671,090,868 (97.53%)	13,125,187,571 (93.64%)	176,379 (0.00%)	8,068,776,013 (57.56%)

## Data Availability

The sequencing data generated in this study have been deposited in the Genome Sequence Archive (GSA) at the National Genomics Data Center (accession numbers CRA024178 and CRA024215), and are publicly accessible at https://ngdc.cncb.ac.cn/gsa (accessed on 27 March 2025).

## Supplementary Materials

The following supporting information can be downloaded at https://www.mdpi.com/article/10.3390/genes16060701/s1: Table S1: Primer sequences used for RT-qPCR; Table S2:The list of differentially expressed mRNAs and miRNA-mRNA target negative correlation; Table S3: The list of differentially expressed miRNAs; Table S4: Table of GO enrichment analysis of target genes of DE miRNAs and DEmRNAs; Table S5: KEGG pathways of target genes of DE miRNAs and DEmRNAs.
